# *Paenibacillus lautus* isolated from the *Sphenophorus levis* gut causes structural and physicochemical changes on polystyrene surface

**DOI:** 10.3389/fmicb.2026.1776542

**Published:** 2026-03-16

**Authors:** Eduardo Pereira de Souza, Henrique Sebestyen-França, Marcos Vinícius Basaglia, Anne Watson, Milene Ferro, Sílvia Helena Prado Bettini, Flávio Henrique-Silva

**Affiliations:** 1 Laboratory of Molecular Biology, Department of Genetics and Evolution, Federal University of São Carlos (UFSCar), São Carlos, Brazil; 2 Department of Materials Engineering, Federal University of São Carlos (UFSCar), São Carlos, Brazil; 3 Department of General and Applied Biology, São Paulo State University (UNESP), Rio Claro, Brazil

**Keywords:** biodegradation, bioremediation, gut bacteria, insect larvae, plastic waste, sugarcane weevil

## Abstract

**Introduction:**

Polystyrene (PS) is a petroleum-based polymer with a recalcitrant structure. Along with the increasing demand, its accumulation in the environment is evident, leading to societal and ecosystemic issues. Therefore, research has focused on strategies for PS biodegradation. Certain insect larvae have been reported to use PS as a carbon source, which depends on their gut microbiota. In this context, we investigated the biodegradation ability of bacteria from the gut of the *Sphenophorus levis* larvae.

**Methods:**

Larvae of *S. levis* were fed expanded polystyrene. Their gut contents were inoculated into a carbon-free liquid medium with PS films. The enriched bacteria were isolated and identified by 16S rRNA gene sequencing. The bacteria were cultured on a carbon-free solid medium in contact with a PS film. After, the films were analyzed by Scanning Electron Microscopy (SEM), Energy-Dispersive X-ray Spectroscopy (EDS), and Fourier Transform Infrared Spectroscopy (FT-IR). Molecular weight changes were assessed by gel permeation chromatography (GPC) using PS films incubated in liquid medium. Whole-genome sequencing of total bacterial DNA was performed using the Illumina platform.

**Results:**

From the five strains isolated from the enrichment, a *Paenibacillus lautus* strain presented the most promising results. Bacteria appeared to be attached to the polymer, altering its topography and forming pits and cracks. Surface oxidation was confirmed by EDS and FT-IR, which detected oxidation-related functional groups, including hydroxyls and carbonyls. The GPC analysis revealed a reduction in the molecular weight of the treated films. Sequencing revealed a 6,950,071 bp genome with an average GC content of 51.11%. Forty contigs were obtained, with 98.9% completeness when compared with the Bacillales ortholog database. Genes encoding putative PS-modifying enzymes, such as peroxidases, ring-cleaving oxygenases, cytochrome P450, and monooxygenases, were identified.

**Discussion:**

The topographical and chemical modifications, together with the changes in molecular weight, provide evidence that the *P. lautus* strain alters the polystyrene structure. Therefore, this strain might be an interesting agent of polystyrene bioremediation, together with its proteins, which will be further studied in the future.

## 1 Introduction

Mitigating the continuous increase in plastic accumulation is one of the major challenges we face. In 2023, 413.8 Mt of plastic was produced, of which 90.4% corresponds to fossil-based materials. Polystyrene (PS) accounted for 5.2% of this amount (Plastics Europe, 2025). PS is a high molecular weight aromatic polymer [(C_8_H_8_)_n_] synthesized from the styrene monomer. The polymer can be solid or foamed and is used in a wide range of applications such as insulation and food packaging (Ho et al., 2018). Despite advantages and applications, the polymer end-of-life is an issue of concern. Recycling is possible; however, it is not economically feasible mainly due to transport costs. On the other hand, incineration results in the release of toxic gases. Hence, most of the discarded PS ends up in landfills or even in natural environments (Geyer et al., 2017; Ho et al., 2018). And, due to the recalcitrant structure of the polymer, they may stay there for a long time (Albertsson and Karlsson, 1993; Artham and Doble, 2008; Ho et al., 2018).

Biodegradation of polystyrene was once considered to be impossible. However, some researchers have put some effort into identifying microorganisms capable of producing chemical and structural changes on the polymer surface. Bacteria and fungi from soil, sea, and even landfills have been isolated and studied in this context. In the last 10 years, it has been observed that some insects with chewing mouthparts are able to digest and mineralize expanded polystyrene (Yang et al., 2015a). Studies with *Tenebrio molitor* revealed that the digestion is intrinsically related to the gut microbiota, which colonize the polymer and induce backbone and topographical changes (Yang et al., 2015b). To date, many insect species have been associated with PS biodegradation, mostly *Lepidoptera* and *Coleoptera* (Cucini et al., 2020; Jiang et al., 2021a; Kim et al., 2020; Wang et al., 2020; Woo et al., 2020).

The sugarcane weevil *Sphenophorus levis* Vaurie (*Coleoptera*: *Curculionidae*) has been a significant sugarcane pest in Brazil since a 1977 outbreak. In the field, the adult female lays eggs in soil near the plant rhizome. After eclosing, larvae penetrate the rhizome and build irregular galleries, living there until they reach adulthood. Larvae, which can reach 15 mm in length, block the rhizomes and basal plant structures, leading to plant death and consequently production loss (Cerda et al., 1999). Our research group has studied *S. levis* biology and molecular control strategies for years. During this time, we noticed that if the larvae were kept inside EPS containers, they would chew the material and escape. Associated with the evidence that insects can digest, mineralize, and even assimilate PS, we decided to evaluate the gut microbes from *S. levis* in the context of polymer biodegradation.

In this study, we fed *S. levis* larvae with expanded polystyrene; their guts were collected, and their contents were cultured in PS-supplemented carbon-free media for enrichment of bacteria possibly involved in PS degradation. A selected bacteria was cultivated in solid media in contact with a PS film. The film surface was evaluated chemically and topographycally, in order to study the effects of the bacteria on the polymer surface. To further investigate the bacterial mechanisms of biodegradation, the complete genome of the isolated bacteria was assessed.

## 2 Materials and methods

### 2.1 Test materials

Expanded polystyrene was kindly provided by Mundi-Eps - Indústria e Comércio de Plásticos LTDA (Brodowski, SP, Brazil; https://www.mundieps.com.br). According to the manufacturer, its density corresponds to 0.014 g/cm3, and neither additives nor catalyzers were added to the polymer. To prepare polystyrene films for microbial degradation, Styrofoam blocks were dissolved in xylene solvent (0.03 g/mL) and placed on 90 × 15 mm glass Petri dishes. After drying for 24 h, the films were immersed in methanol and deionized water.

*S. levis* larvae were purchased from Pragas.com (Piracicaba, SP, Brazil; https://www.pragas.com.vc). The study was registered (no. ADAFBFD) in the Sistema Nacional de Gestão do Patrimônio Genético e do Conhecimento Tradicional Associado (SisGen).

### 2.2 Larvae rearing

Fifteen-day-old larvae were distributed into two different feeding groups of 33 individuals. The insects were fed a sugarcane-based artificial diet (Degaspari et al., 1987) or expanded polystyrene blocks in 150 mg Petri dishes. Larvae were reared individually. They were weighed on the first and the fifth (last) day of the experiment and the larval growth was assessed. The rearing was performed in a Panasonic MLR - 352H chamber under controlled parameters: temperature (26 ± 2 °C), photoperiod (12:12), and relative humidity (55%−65%).

### 2.3 Isolation of putative PS-degrading microorganisms

After 5 days of EPS feeding, the larvae were collected and dissected as previously described (Rinke et al., 2011). First, the specimens were kept on ice for 10 min to immobilize them. They were submerged in 70% (v/v) ethanol for 3 min for surface sterilization, then maintained in a sterile PBS solution (PBS; 137 mM NaCl, 2.7 mM KCl, 10 mM Na_2_HPO_4_, 2 mM KH_2_PO_4_, pH 7.4) before dissection. Larvae heads were removed using scalpels, and the guts were pulled off from the body with tweezers. The collected intestines, in pools of 10, were immersed in PBS and vortexed for 5 min. The material was decanted on ice for 20 min, and the supernatant was collected.

The supernatant was inoculated in 100 mL of LCFBM (Liquid Carbon Free Basal Media) containing 1 g of polystyrene films (3 mm × 3 mm × 80 μm). The carbon free media is from the American Society for Testing and Materials (ASTM) Standard Practice for Determining Resistance of Plastics to Bacteria (ASTM G22–76). It is composed by KH_2_PO_4_ (0.7 g/L), K_2_HPO_4_ (0.7 g/L), MgSO_4_·7H_2_O (0.7 g/L), NH_4_NO_3_ (1.0 g/L), NaCl (0.005 g/L), FeSO_4_·7H_2_O (0.002 g/L), ZnSO_4_·7H_2_O (0.002 g/L), and MnSO_4_·H_2_O (0.001 g/L). The culture was incubated at 24 °C and 120 rpm. After 60 days, the PS films were collected and vortexed for 5 min to release the bacteria. Then, they were plated on LB agar medium and incubated at 30 °C.

According to their morphology, different colonies were selected and re-streaked. Total bacterial DNA was extracted using the NucleoSpin Microbial DNA (Macherey-Nagel) kit. In order to identify the bacteria, the 16S region was amplified by PCR [(primers: 27F 5′-AGAGTTTGATCMTGGCTCAG3′) and 1492R 5′-GGTTACCTTGTTACGACTT3′)] (Weisburg et al., 1991). The amplicons were sequenced using the dideoxy method (Sanger et al., 1977) and the V3-V4 primer pair: V3-V4: 314F (5′ CCTACGGGNGGCWGCAG 3′) and 805R (5′GACTACHVGGGTATCTAATCC3′). Sanger sequencing was performed with BigDye Terminator v3.1 Cycle Sequencing Kit (Applied Biosystems) and analyzed in a 3130XL Genetic Analyzer (Applied Biosystems).

### 2.4 Evaluation of the bacteria-polystyrene interaction

First, bacteria were cultivated in LB medium (200 rpm, 30 °C) until they reached the log phase. The cultures were centrifuged for 7 min at 2,700 × g. The biomass was resuspended in saline water. The centrifugation process was repeated three times to wash the pellet. Finally, the material was resuspended in LCFBM and spread onto a plate with the same medium supplemented with agarose. A polystyrene film was laid above the bacteria. The culture was incubated for 60 days at 24 °C, when the films were removed and subjected to evaluation.

To analyze the bacterial proliferation and morphology on the polymer, we examined the films by Scanning Electron Microscopy (SEM). For structural and chemical post-degradation evaluation, films were washed with 2% SDS overnight to remove microorganisms. Film metallization with gold was carried out in Balzers Sputter Coater SCD 004. Images were captured in a Magellan scanning electron microscope. The same films were analyzed using a TESCAN MIRA microscope to investigate their surface elemental composition through energy-dispersive X-ray spectroscopy. To identify the functional groups formed on the polymer surface after exposure to bacteria, we performed Fourier Transform Infrared Spectroscopy (FTIR-ATR) using Nicolet 6700 (Thermo Scientific) equipment. In order to compare the hydrophobicity of the control PS and the bacteria-treated films, a Water Contact Angle (WCA) analysis was carried out using Attension Theta Flex (Biolin Scientific, Gothenburg, Sweden) equipment at 24 °C. The Welch two-sample *t*-test (*n* = 3) was used to compare the treated films to the control.

Bacteria were also cultivated individually in liquid media. Bacterial cells were prepared as described and resuspended in 45 mL of LCFBM (OD_600_ = 0.1). A 30 × 30 mm film of 30 mg of weight was included. After 60 days, the weight was measured. To assess the molecular mass shift in the films, Gel Permeation Chromatography (GPC) was conducted. Molar mass analysis was performed in a Shimadzu (Kyoto, Japan) Prominence GPC system equipped with a Phenogel pre-column, 5 μm, and two columns in series: Phenogel 5 μm, 1 × 10^6^ Å and 5 μm, 1 × 10^4^ Å (all from Phenomenex, Torrance, CA, USA). The sample volume was 50 μL (1 mg mL^−1^), and the analysis was performed at 35 °C. A differential refractive index detector (Shimadzu RID-10A) was used. The mobile phase was THF containing 0.3% triethylamine at a flow rate of 0.8 mL min^−1^. The system was calibrated with polymethylmethacrylate (PMMA) standards (EasyCal, Sigma-Aldrich) (Mp ~ 800 −2,000,000 g mol^−1^). All molecular masses reported are, therefore, relative to PMMA standards.

### 2.5 Genome sequencing and analysis

Bacterial genomic DNA was sequenced using high-throughput sequencing. Libraries were prepared with the Illumina DNA Prep kit and quantified by qPCR. Paired-end sequencing (2 × 100 bp) was performed on an Illumina Nextseq 2000 (Illumina, Inc., San Diego, CA, USA) and analyzed with BCL Convert software, which generated sequences in fastq format accompanied by the Phred score (Ewing et al., 1998). The raw reads evaluation was carried out with the FastQC software (0.12.1) (https://www.bioinformatics.babraham.ac.uk/projects/fastqc/).

Preprocessing and assembly were done with Shovill 1.1.0 version (https://github.com/tseemann/shovill) pipeline. This pipeline includes bioinformatics tools such as Trimmomatic (Bolger et al., 2014) to remove adapters, Lighter 1.1.3 version (Song et al., 2014), SPAdes 4.0.0 version (Bankevich et al., 2012) for assembling the draft genome, BWA 0.7.17 version (Li et al., 2009) for reads mapping into draft genome and Pilon 1.24 version (Walker et al., 2014) that corrects inaccuracies in the assembly. Contigs smaller than 500 bp were removed.

The genome quality was evaluated with BUSCO (5.8.0 version) (Manni et al., 2021). This software evaluates genome quality based on the presence of an expected group of single-copy conserved genes. These evaluation groups are derived from the OrthoDB (version 10) database (Kriventseva et al., 2019). As references, ortholog groups from the order Bacillales were used, in reference to the previous genus identification by Sanger sequencing. Taxonomic classification of the genomes was conducted with the GTDB-Tk version 2.4.0 pipeline (Chaumeil et al., 2022).

A comprehensive genomic investigation was carried out, focusing on key elements in plastic degradation. Annotation was carried out with Bakta version 1.9.4. Bakta is a prokaryotic annotation pipeline that employs a diverse range of programs and databases to accurately annotate prokaryotic genomes from diverse taxonomic origins (Schwengers et al., 2021). To functionally characterize the genome of the studied strain, the sequences were analyzed with Gene Ontology and classified according to Enzyme Commission Numbers in the Omicsbox platform (BioBam Bioinformatics, 2019).

### 2.6 Data availability

The raw sequence data are available in the Short Read Archive (SRA) GenBank database: BioProject (PRJNA1373219), BioSample (SAMN53646917), and SRA (SRR36290426).

## 3 Results

### 3.1 Larvae growth and survival

**Figure 1:**
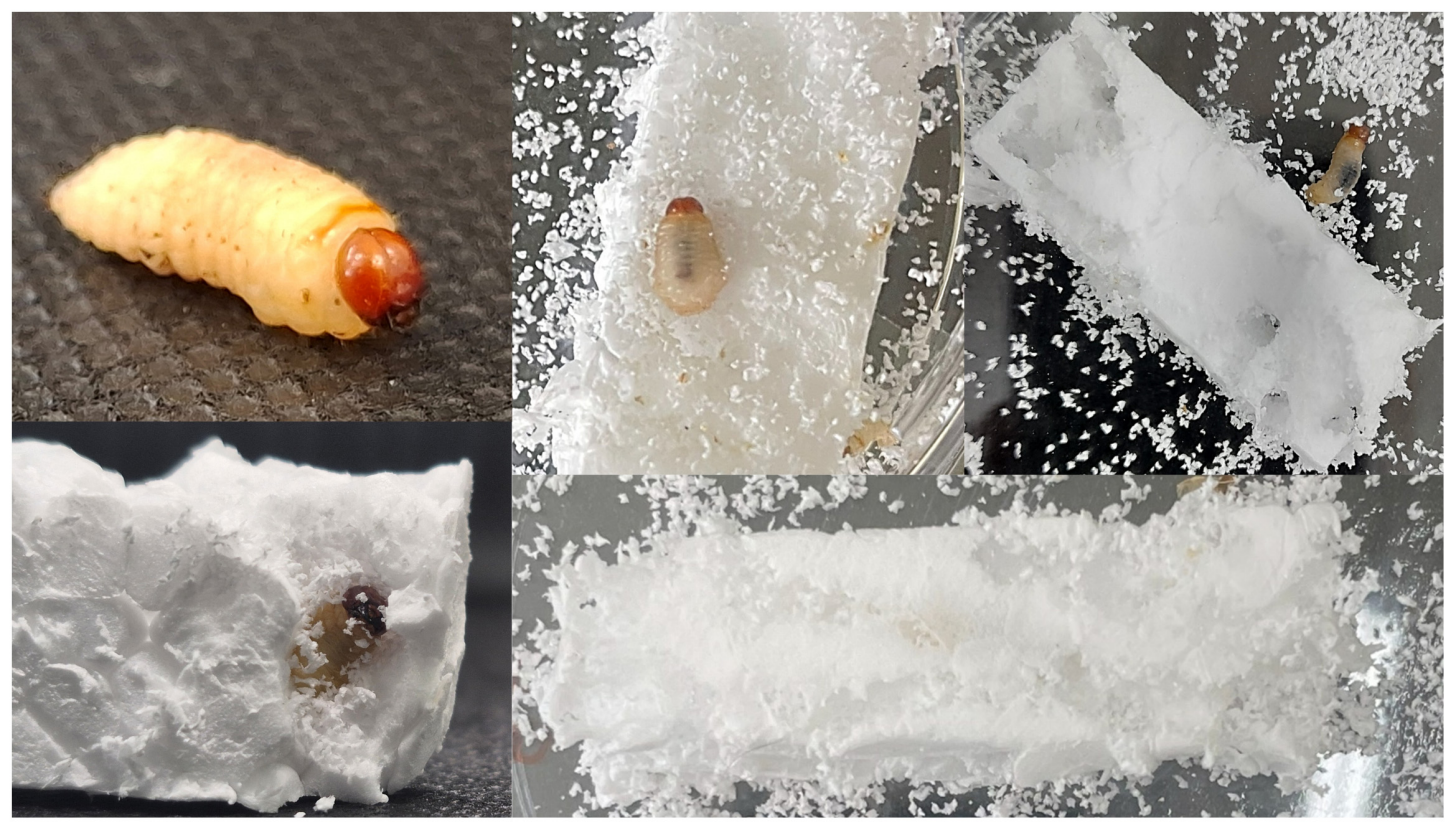
Mosaic with photographs demonstrating the interaction of the *S. levis* larvae with EPS blocks.

*Sphenophorus levis* larvae were reared under controlled environmental conditions with feeding restricted to expanded polystyrene blocks. The larvae seemed to mimic their behavior in the field when colonizing sugarcane plants. They chewed the EPS blocks as they formed galleries (Figure 1; Supplementary Video 1). After 5 days, the larvae were weighed. Those fed on EPS showed a mass reduction to 44.9% ± 9.9% of their original weight, likely due to the consumption of energy reserves, while larvae on the artificial diet either maintained their weight or experienced a slight positive or negative change (9.6% ± 47.6% of initial weight). Larvae consumed EPS at a mean of 6.0 ± 3.8 mg.

### 3.2 Isolation and identification of putative PS-degrading bacteria

The gut content of EPS-fed larvae was extracted and inoculated in LCFBM media containing PS films as the sole carbon source. After 60 days of culture, the medium became turbid, and biofilms were evident on the polymer surface. The microorganism solution was then spread on a LB agar plate. Bacteria grew and were distinguished by their colony morphology. Five microorganisms were isolated and identified by genus with V3-V4 region sequencing: *Bacillus* sp., *Lysinibacillus* sp., *Paenibacillus* sp., *Cytobacillus* sp. and *Raoultella* sp. (NCBI GenBank Accession numbers: PX753250, PX753251, PX753252 PX753253, PX753254, respectively). The ability of bacteria to modify PS was assessed, and, in line with preliminary results, we focus here on the *Paenibacillus* strain. Bacteria such as *Bacillus* sp., *Cytobacillus* sp., and *Raoultella* sp. were able to modify the surface topography; however, no changes in surface functional groups were observed. No modifications were observed for the *Lysinibacillus* sp. strain.

### 3.3 Polystyrene films evaluation

**Figure 2:**
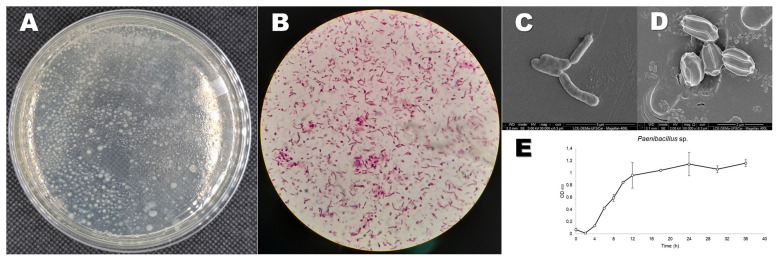
*Paenibacillus lautus* from *S. levis* isolated from the enrichment in LCFBM media with PS. **(A)** Bacterial growth in LB agar media. **(B)** Gram-stained bacteria under light microscope. **(C)** Electron micrography of vegetative bacterial cells. **(D)** Electron micrography of endospores. **(E)** Growth curve (Optical Density x Time) of *Paenibacillus lautus* growing in LB media (200 rpm, 30 °C).

**Figure 3:**
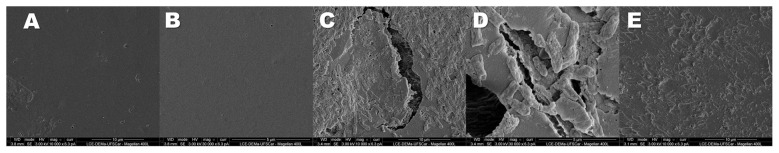
Surface electron microscopy of polystyrene films. **(A, B)** Control PS films. **(C, D)** *P. lautus* treated films. **(E)** *P. lautus* washed treated film. The micrographs show pits, cracks, and other surface modifications as well as bacterial proliferation.

**Figure 4:**
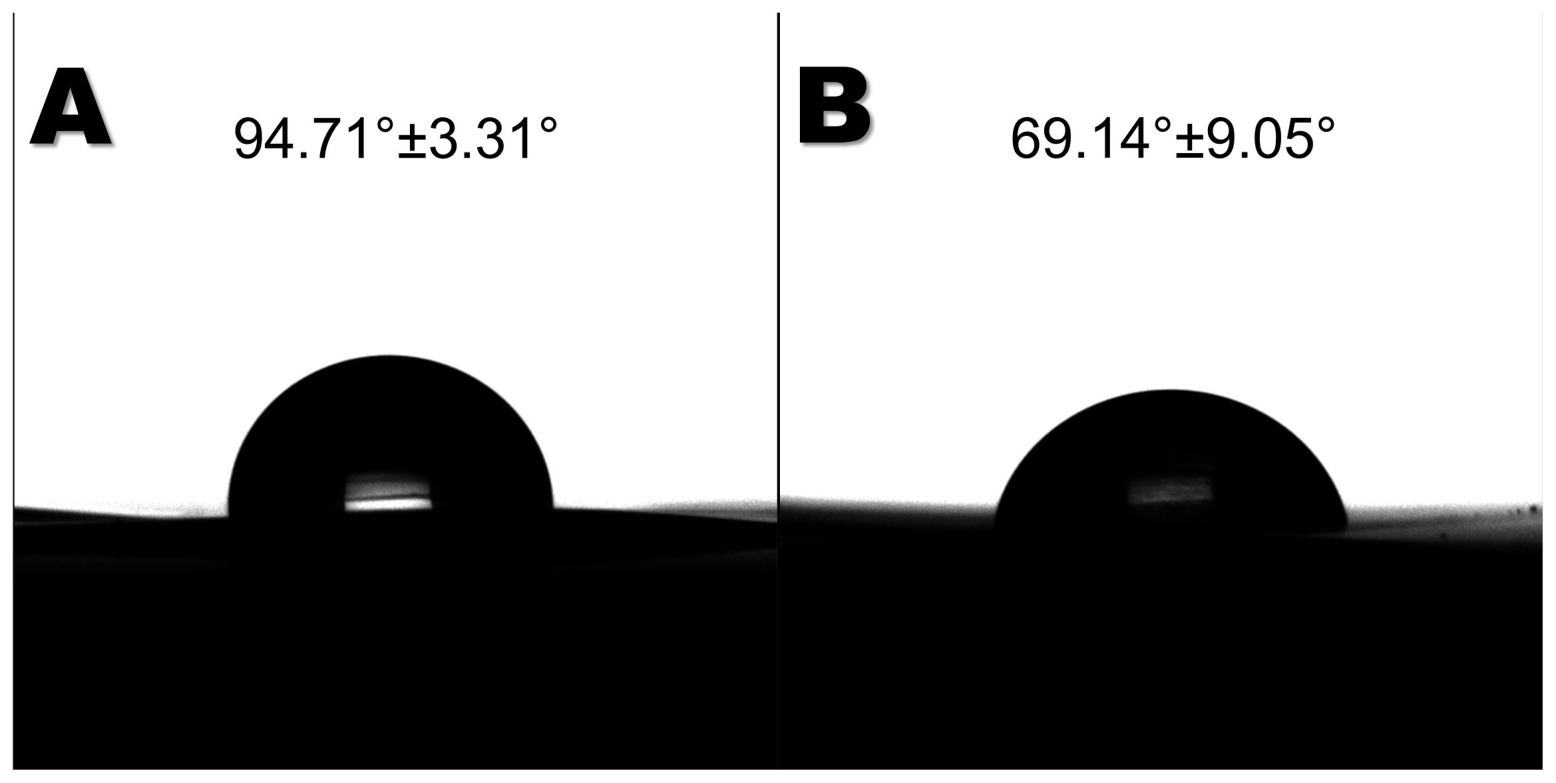
Contact angle analysis. **(A)** Control PS-film. **(B)** Bacteria-treated film. Bacteria changed the hydrophilicity of the de PS films.

*Paenibacillus* sp. (Figure 2) was grown in LB medium until the log phase and collected. They were spread on a solid Carbon Free Basal Media plate and covered with a PS film. After 60 days, the PS films were analyzed by Scanning Electron Microscopy (Figure 3). In the control sample, the films appeared smooth and free of any major defects. In the treated samples, the bacteria seemed to accumulate on the surface and interact with the polymer. In the post-culture-washed samples, it is noticeable that some structures, such as pits, cavities, and other structural changes, were formed. The hydrophobicity of the PS films was also changed under bacterial action. The WCA measurement indicated that the observed angle for the control was 95.71° ± 3.31° while the bacteria-grown films were 69.14° ± 9.05° (Figure 4) showing a statistically significant difference (*p* < 0.05). This reveals that the *Paenibacillus* sp. strain can lead to a more hydrophilic PS film.

**Figure 5:**
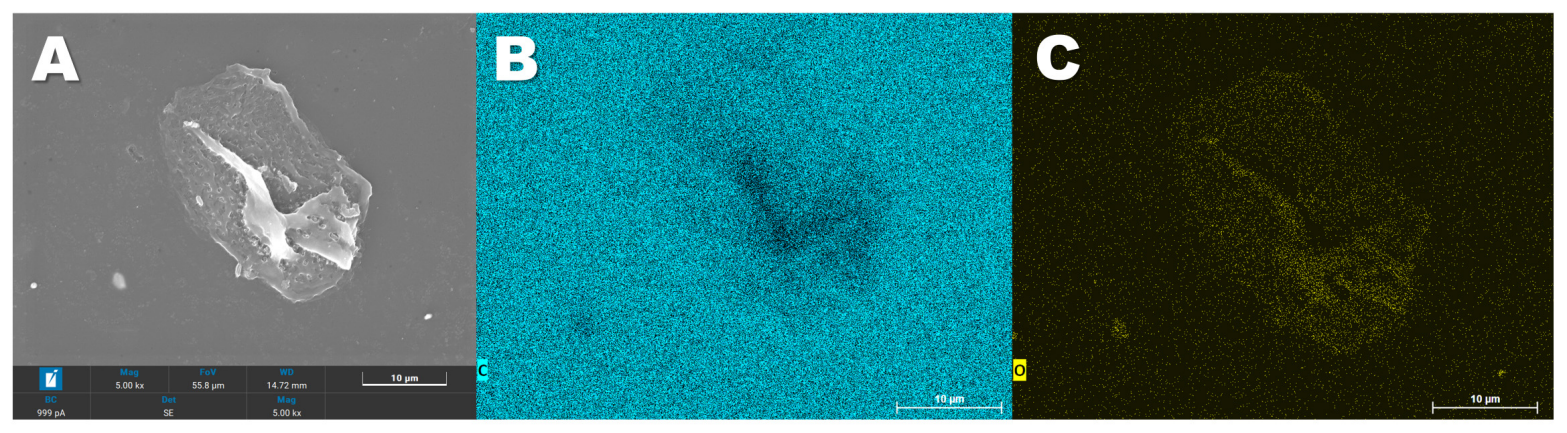
Energy dispersive spectroscopy of polystyrene films. **(A)** Electron micrograph of the region of analysis. **(B)** Distribution of carbon atoms. **(C)** Distribution of oxygen atoms.

**Figure 6:**
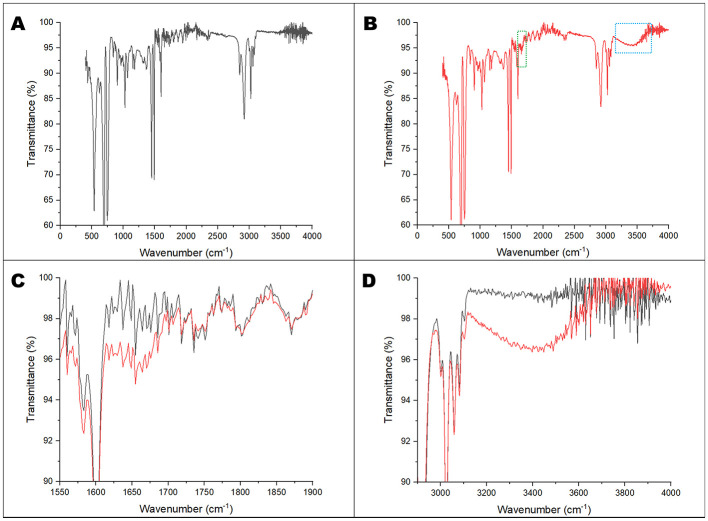
FTIR-ATR analysis of PS films in contact with *Paenibacillus*. **(A)** Control, in black. **(B)** Bacteria-treated PS, in red. The expanded region in green **(C)** highlights the formation of C=C or C=O groups and the region in blue **(D)** corresponds to the hydroxyl (-OH) group.

The same films were submitted to energy-dispersive X-ray spectroscopy mapping in order to evaluate the changes in the chemical structure of the polymer (Figure 5). It was clear that in the regions affected by the microbial action, oxygen atoms (which are not originally present in the PS structure), represented in yellow, accumulate, in contrast to carbon, represented in blue. FTIR-ATR analysis of the film surface was implemented to assess the formation of functional groups after bacterial activity (Figure 6). The generated spectra for the *Paenibacillus-*treated films revealed a protuberance extending from 3,100 to 3,600 cm^−1^ wavenumber, associated with the formation of hydroxyl groups. There is also a peak at 1,600–1,680 cm^−1^ regions. Changes in this region of the spectra may be correlated to C=C (1,600–1,680 cm^−1^) or C=O (1,650–1,760 cm^−1^) bonds (Silverstein et al., 2005). Altogether with the EDS data, the FTIR-ATR provided evidence of surface chemical modifications that may lead to biodegradation.

**Figure 7:**
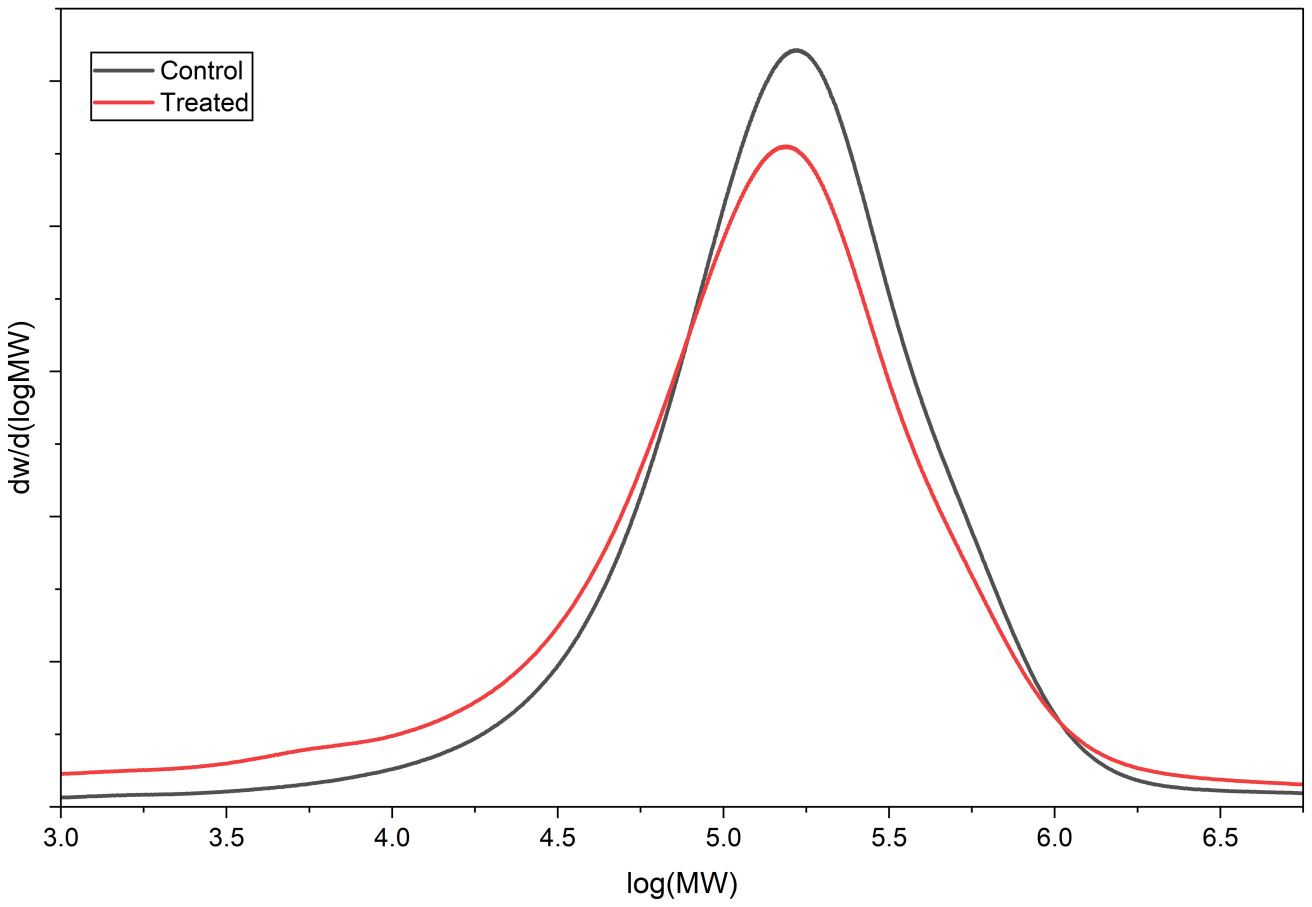
GPC analysis of PS films in contact with *P. lautus* for 60 days. The figure presents the molecular weight distribution of a PS film, as measured by GPC, 60 days in contact with the bacteria in contrast to the control.

The films were incubated in liquid media with bacteria for 60 days. The mass loss accounted for 3.73% ± 0.57% of the total polymer input into the culture media. The same films were investigated regarding changes in the molecular weight. Figure 7 presents the molecular weight distribution of a PS film, as measured by GPC, after 60 days in contact with the bacteria in contrast to the control. The distribution shifts toward lower molecular weight, indicating that chain degradation occurred in the presence of bacteria. It is worth pointing out that the bacteria probably act only on the surface of the film, although the whole film was dissolved in the mobile phase for GPC, so that the vast majority of the mass analyzed comes from the core, not from the surface, where the main decrease in the average molar mass is expected, due to degradation by the bacteria. Taking this into account, the shift in average molecular weight is expected to be small, although it is observable.

### 3.4 Whole genome identification characterization

In the face of the changes observed on the polymer surface under the action of the bacteria, understanding the molecular mechanisms associated with this event is relevant. That is why we investigated the total genome of the microorganism through sequencing to identify candidate genes related to plastic degradation. Illumina sequencing produced a total of 27,160,152 paired reads. Table 1 presents the assembly statistics of the genome. The assembly resulted in a singular circular chromosome of 6,950,071 base pairs in size with an average GC content of 51.11%. Forty contigs were obtained. Genome quality analysis was performed with BUSCO against the Bacillales Orthologs database. 98.9% of the genes are complete (single copy or duplicated), 0.5% are fragmented, and 0.6% are missing. The strain was identified as *Paenibacillus lautus* (ANI: 96.8%).

**Table 1:** Assembled genome metrics.

**Sample**	**Total size**	**Longest contig**	**# contigs**	**N50**	**L50**	**auN**	**%GC**
*P. lautus*	6,950,071	796,201	40	402,956	6	412,199	51.11

The genomes were annotated with the Bakta pipeline, and the obtained results are displayed in Table 2. Of 6,675 putative genes, 6,260 correspond to coding regions of recognized proteins. To further analyze these coding regions, we classified them according to Gene Ontology (Supplementary Figure 1) and Enzyme Commission Numbers (Supplementary Figure 2). We placed particular emphasis on oxidoreductases and hydrolases, which have been described as relevant in plastic biodegradation. Based on the E.C. numbers classification, 561 sequences correspond to oxidoreductases and 1,241 to hydrolases.

**Table 2:** Genome annotation.

**Sequence classification**	**Number of sequences**
tRNAs	44
tmRNAs	1
rRNAs	3
ncRNAs	11
ncRNA regions	53
CDSs	6,260
Pseudogenes	24
Hypotheticals	273
sORFs	4
oriCs	1
signal peptides	0
Gaps	0
oriVs	0
oriTs	0

The data correlates the classification and the number of the respective sequences.

Among the annotated genes, the bacterial genome presents a wide range of oxidoreductases, important for the initial steps of polymer modification. Cytochrome P450, Multicopper oxidases, monooxygenases, ring-cleaving dioxygenases, and glutathione peroxidase are examples of putative actors in PS surface changes.

## 4 Discussion

Larvae of *S. levis* were subjected to a restricted diet consisting of expanded polystyrene, aiming to alter the community structure in the gut and promote the growth of PS-degrading related microorganisms. A culture enrichment was performed with the gut contents in a minimal medium with polystyrene as the sole carbon source. One of the five isolated strains was identified as *Paenibacillus lautus*, which, according to preliminary results, was considered for further investigation.

*P. lautus* is a gram-positive spore-forming rod-shaped bacterium generally found in clinical samples, water, and soil. Notably, some research has revealed that these species are present in the rhizosphere of sugarcane plants as nitrogen-fixing agents (Singh et al., 2020). Furthermore, they have been described as endophytic bacteria important in disease control (Jayakumar et al., 2019). Since sugarcane is the main nutritional source for *S. levis*, even in artificial diets, the consumption of such material may have led to the establishment of *P. lautus* in the larval gut. This observation aligns with previous reports; it is not the first time a *Paenibacillus* strain was isolated from *S. levis*. Rinke et al. (2011) obtained the microorganism from laboratory-reared larvae and assessed its lignocellulolytic ability (Rinke et al., 2011).

In the current literature, there are just a few reports of members of the *Paenibacillaceae* family associated with plastic degradation. A strain isolated from a landfill was able to degrade LDPE; in this case, surface functionalization, bond scission, and weight loss were observed. These events are probably related to an alkane hydroxylase (AlkB) (Bardají et al., 2019). In another instance, a soil-obtained *Paenibacillus urinalis* presented evidence of polystyrene degradation, which was confirmed by HPLC degradation products analysis (Atiq et al., 2010).

*P. lautus* from the sugarcane weevil larvae gut was tested against polystyrene films to evaluate the ability to modify the polymer surface. The bacteria seemed to interact with the PS films, leading to topographical changes such as pits and cavities, as described for other organisms such as *Pseudomonas* sp. and *Exiguobacterium* sp. (Kim et al., 2020; Yang et al., 2015b). In contrast, the control films remained smooth and without any defects. Furthermore, the exposure of the films to *P. lautus* led to a reduction in hydrophobicity, as observed by water contact angle analysis. Since the more hydrophilic the substrate is, the easier it is for bacteria to attach and interact, this facilitates the next steps of biodegradation (Jiang et al., 2021b; Yang et al., 2015b). In addition, the microbial activity also led to modifications in the surface chemical composition. Energy-dispersive X-ray spectroscopy mapping revealed an accumulation of oxygen atoms over the bacteria-grown PS films. This phenomenon is related to the oxidative process mediated by the bacteria. As a result, the surface becomes more hydrophilic and susceptible to nucleophilic attack, leading to polymer breakage. This event was also observed for *Pseudomonas* sp. and *Massilia* sp. strains (Jiang et al., 2021b; Kim et al., 2020). FT-IR analysis described the formation of oxygen-related functional groups. The first step in polystyrene biodegradation is the hydroxylation of the polymeric structure. The 3,100–3,600 cm^−1^ region is associated with the stretching vibration of the O-H in the alcohols and phenols (Silverstein et al., 2005). Likewise, similar graphical structures with the same magnitude were observed for PS films in contact with *Pseudomonas* sp., *Enterobacter* sp., *Klebsiella* sp., or *Cellulosimicrobium* sp. (Kang et al., 2023; Kim et al., 2020; Lin et al., 2024). Additionally, the absorption peak around the 1,600–1,680 cm^−1^ region might be correlated to C=C (1,600–1,680 cm^−1^) or C=O (1,650–1,760 cm^−1^) bonds. Lin et al. (2024) observed the same pattern for *Klebsiella* sp. or *Cellulosimicrobium* sp. Altogether, this reveals that the polymer underwent further oxidation, which may culminate in bond breaking. Although slight, PS depolymerization was evident in the GPC assay. The molecular size shift indicates a decrease in the molecular weight distribution, suggesting cleavage/depolymerization of the long-chain structure of the polymer and the formation of smaller PS fragments, as observed for *Exiguobacterium* sp. (Yang et al., 2015b) and *Acinetobacter* sp. (Wang et al., 2020).

The genome of the isolated bacteria provides evidence of proteins that may be involved in this process. The implementation of hydroxyls and carbonyls on the polymer chemical structure is intrinsically associated with oxygenases and related proteins. The Cytochrome P450 superfamily comprises heme-containing oxygenases related to diverse catalytic roles (Greule et al., 2018; Nelson, 2018). Although the hydroxylation of C-H bonds is the best characterized function of the counterparts of this superfamily, there is evidence of a wide range of reactions such as aromatic oxidation, alkene epoxidation, and heteroatom oxidation (Greule et al., 2018; Guengerich and Munro, 2013; Zhang and Li, 2017). Due to the variety of oxidation reactions, this superfamily is considered crucial for polymer degradation (Yeom et al., 2022). The isolated *P. lautus* strain displays different Cytochrome P450 genes: the bifunctional CYP102, CYP106, and CYP107. CYP102 is well known for its role in the oxidation of medium-chain fatty acids. Some studies have shown that the well-characterized CYP102 from *Priestia megaterium* (formerly *Bacillus megaterium*) has low alkane hydroxylase activity; however, site-directed mutations improved the reactivity toward n-alkane substrates (Feenstra et al., 2007; Weber et al., 2011). On the other hand, it was observed that wild-type CYP102 from *Bacillus thuringiensis* was the key element for polyethylene modification by oxidation. The respective transcript was upregulated (along with CYP106 and CYP107, also present in the *P. lautus* strain genome) and functional assays confirmed that it was crucial for oxygen incorporation (Tarara et al., 2025; Yun et al., 2023). Furthermore, CYP102 has an effective role in the oxidation of styrene. The recombinantly produced enzyme from *Priestia megaterium* is able to catalyze the epoxidation of the molecule *in vitro* (Eiben et al., 2006; Huang et al., 2011; Yuan et al., 2009). These are indicatives that this enzyme may be associated with the main chain degradation of polystyrene and in processing the released styrene monomers.

Glutathione peroxidases (GPxs) were also described as important actors in plastic degradation (Rong et al., 2024). This group of enzymes catalyzes the reduction of hydrogen peroxide while glutathione (GSH) acts as the reducing agent (Bao et al., 2023). Two gene sequences have been found in the *P. lautus* genome. Rong et al. (2024) proposed a mechanism for polyethylene biodegradation mediated by a *Rhodococcus* sp. glutathione peroxidase. They described that, in cooperation with its superoxyanion radicals, the glutathione peroxidases were able to trigger the depolymerization of (LDPE). Like PE, PS presents a CC backbone. We hypothesize that, under similar conditions, PS oxidation might be triggered by GPxs activity, although further investigation is required.

The isolated *P. lautus* strain presents a collection of enzymes related to the processing of aromatic compounds. Some enzymes can act specifically in the polystyrene side-chain cleavage pathways. Aromatic ring hydroxylases are proposed to initiate polymer modification on the PS phenyl groups and may also contribute to the oxidation of styrene and other aromatic compounds after the PS decomposition (Hou and Majumder, 2021). Ring-cleaving dioxygenases have important roles in the degradation of aromatic compounds by bacteria. They add simultaneously two oxygen atoms to the ring (Fetzner, 2012). Polystyrene biodegradation studies involving *Exiguobacterium* sp. indicated that this process may be initiated via oxidation of the aromatic rings by a ring-cleaving dioxygenase (Parthasarathy et al., 2022).

The PS modifications promoted by the proteins of the *P. lautus* strain begin with oxidation steps. Oxidation of the main chain may be triggered by the action of glutathione peroxidases and by the insertion of hydroxyls by monooxygenases such as the Cytochrome P450 102. On the other hand, aromatic ring oxygenases may play an important role in changes to side-chain functional groups. Further oxidation steps (e.g., alcohol oxidase), introducing C=O bonds, may provide a more readily hydrolyzable substrate for hydrolases. This would result in the molecular mass shift and the formation of pits and cracks on the surface of the polymer. To confirm the importance of these enzymes, expression quantification analysis and activity assays are mandatory and will be conducted in the following steps.

## 5 Conclusion

The gut of the sugarcane weevil is a promising reservoir of microorganisms of biotechnological interest. In this study, it was possible to isolate a *Paenibacillus lautus* strain capable of modifying the surface of polystyrene films. Bacteria grew over the films, which caused modification of the surface topography and increased the substrate's hydrophilicity through the insertion of oxygen-related functional groups via oxidation. Also, a molecular mass shift was observed indicating the formation of shorten chain size polymers. To further explore this process, the genome from this strain was assessed, revealing potential genes related to polystyrene biodegradation, such as cytochrome P450, glutathione peroxidases, and aromatic-ring oxidases, as well as a wide range of other monooxygenases and hydrolases. To further comprehend the role of these proteins in PS modification, some additional data such as gene expression, protein function and analysis of the polymer degradation products should be assessed. Therefore, the observed interaction between *P. lautus* and polystyrene, combined with whole genome sequencing data, may enable the development of novel biotechnological approaches for plastic bioremediation.

## Data Availability

The datasets presented in this study can be found in online repositories. The names of the repository/repositories and accession number(s) can be found below: https://www.ncbi.nlm.nih.gov/, SRR36290426; https://www.ncbi.nlm.nih.gov/genbank/, PX753250; https://www.ncbi.nlm.nih.gov/genbank/, PX753251; https://www.ncbi.nlm.nih.gov/genbank/, PX753252; https://www.ncbi.nlm.nih.gov/genbank/, PX753253; and https://www.ncbi.nlm.nih.gov/genbank/, PX753254.
