# Supplementary material for: Paenibacillus lautus isolated from the Sphenophorus levis gut causes structural and physicochemical changes on polystyrene surface
Source: Front Microbiol. 2026 Mar 16;17:1776542. doi: 10.3389/fmicb.2026.1776542 (PMC13033606; doi:10.3389/fmicb.2026.1776542)
Supplement: Supplementary file 1 [file Table_1.docx]

Supplementary Material

## Supplementary Figures


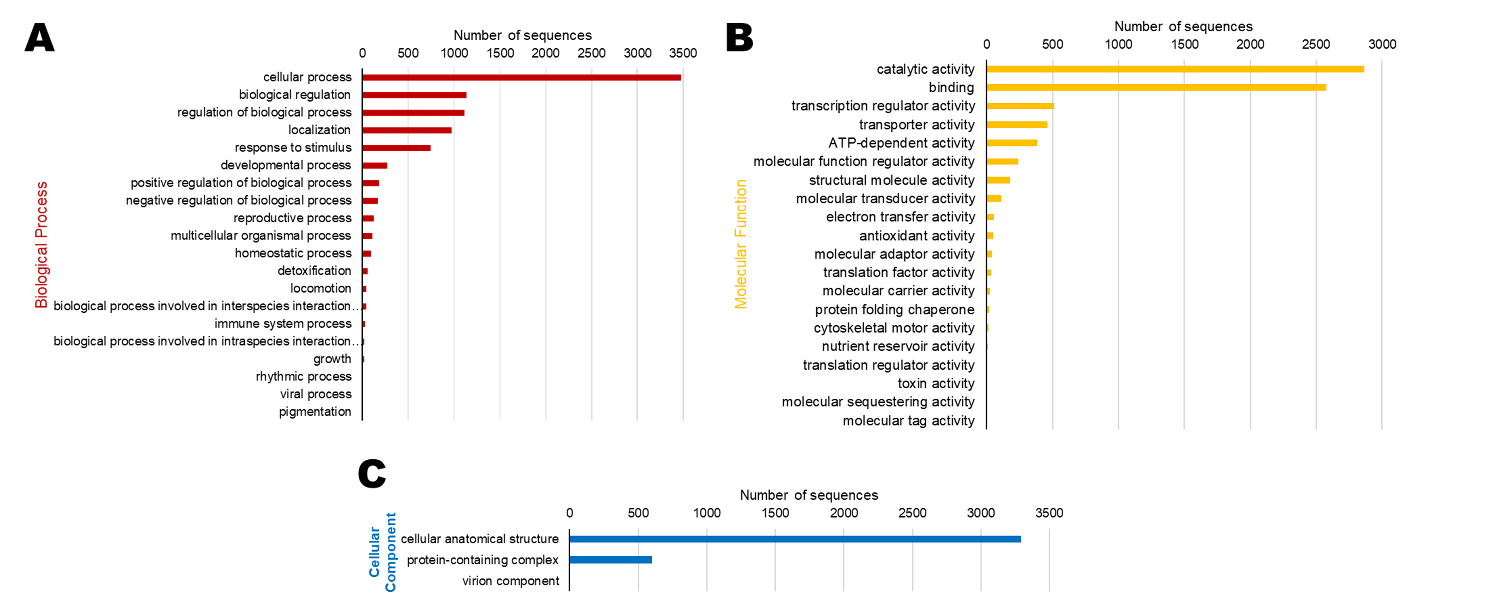


**Supplementary Figure 1.** **Gene Ontology Terms attribution for *Paenibacillus lautus* sequences.** **(A)** Biological Process. **(B)** Molecular Function. **(C)** Cellular Component.


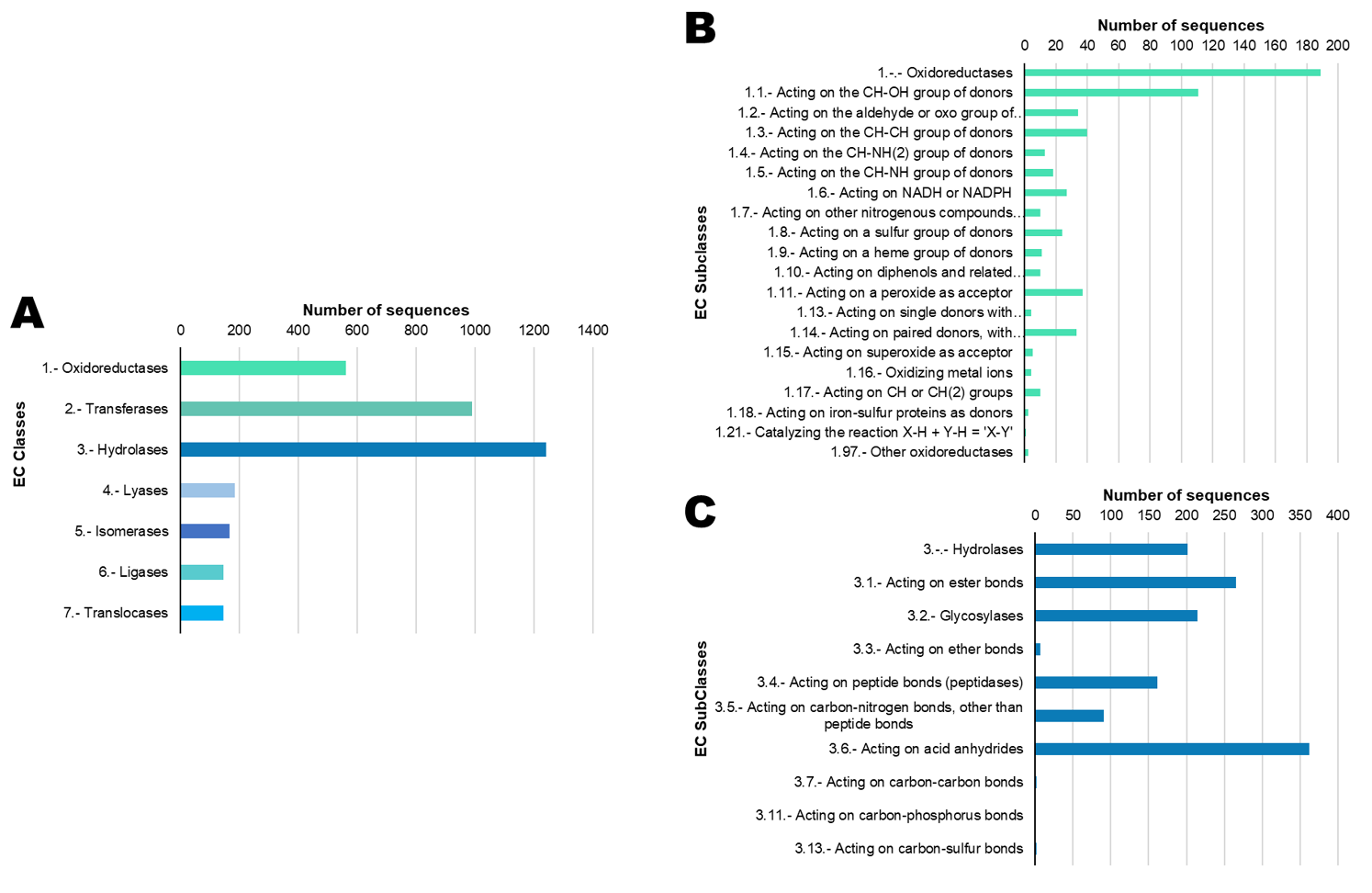
**Supplementary Figure 2.** **E.C. numbers distribution for the annotated genes from *P. lautus.* (A)** Classes distribution. **(B)** Oxidoreductases subclasses distribution **(C)** Hydrolases subclasses distribution.
